# Apolipoprotein E selectively supports gammaherpesvirus replication in macrophages

**DOI:** 10.1128/jvi.00480-25

**Published:** 2025-05-29

**Authors:** Damon L. Schmalzriedt, Carlie A. Aurubin, Cade R. Rahlf, Matthew A. Brown, Jordan M. Bobek, Philip T. Lange, Xander G. Bradeen, Daisy Sahoo, Vera L. Tarakanova

**Affiliations:** 1 Department of Microbiology and Immunology, Medical College of Wisconsin 735651, Milwaukee, Wisconsin, USA; 2 Medical Scientist Training Program, Medical College of Wisconsin 5506, Milwaukee, Wisconsin, USA; 3 Department of Biochemistry, Medical College of Wisconsin 166012, Milwaukee, Wisconsin, USA; 4 Department of Medicine, Medical College of Wisconsin 196253, Milwaukee, Wisconsin, USA; 5 Cardiovascular Center, Medical College of Wisconsin 601983, Milwaukee, Wisconsin, USA; 6 Cancer Center, Medical College of Wisconsin 166013, Milwaukee, Wisconsin, USA; University of Virginia, Charlottesville, Virginia, USA

**Keywords:** gammaherpesvirus, macrophage, apolipoprotein E, lytic replication

## Abstract

Gammaherpesviruses establish lifelong infections in over 90% of adults worldwide and contribute to the development of several cancers. Endogenous lipid synthesis pathways support lytic and latent life cycles of several gammaherpesviruses. However, the role of circulating lipoproteins and the corresponding apolipoproteins in gammaherpesvirus infection remains unknown. Apolipoprotein E (ApoE) is a protein that associates with lipoproteins in circulation and supports bidirectional lipid transport to maintain lipid homeostasis. Interestingly, ApoE differentially affects several virus families, but its role in gammaherpesvirus infection has not been evaluated. In this study, we demonstrate that ApoE expression was increased in murine gammaherpesvirus 68 (MHV68)-infected macrophages in a type-I interferon (IFN)-dependent manner. Intriguingly, ApoE expression was usurped to support MHV68 lytic replication and expression of lytic viral genes. The proviral effects of ApoE in macrophages were independent of the conventional functions of ApoE in the regulation of endogenous lipid synthesis and type I IFN signaling. Finally, the proviral effects of ApoE were limited to the lytic life cycle, as the establishment of MHV68 latency in macrophages was not altered by the ApoE genotype of chronically infected mice. Thus, our study defines a viral life cycle-specific proviral role of ApoE in gammaherpesvirus infection.

**IMPORTANCE:**

ApoE is an apolipoprotein that mediates lipid transport and exchange between tissues and the circulation. ApoE differentially affects several virus families, but its role in gammaherpesvirus infection remains unknown. Here, we show that ApoE supported lytic gammaherpesvirus replication in primary macrophages and that infected macrophages increased expression of ApoE in an interferon-dependent manner. However, ApoE expression did not affect viral latency *in vivo*, implying a novel viral life cycle-specific proviral role for ApoE in gammaherpesvirus infection.

## INTRODUCTION

Gammaherpesviruses are highly prevalent pathogens that establish lifelong infections and are associated with several types of malignancies. Because increased viral reactivation and lytic replication contribute to gammaherpesvirus-driven cancer (1–7), it is critical to understand host factors that regulate the gammaherpesvirus lytic cycle.

Host lipid metabolism has become an important regulator of lytic and latent gammaherpesvirus infection. The published studies to date have focused on endogenous lipid synthesis pathways and their largely proviral role in supporting gammaherpesvirus replication and latency (8–16). Importantly, cellular lipid homeostasis is maintained by the combination of endogenous lipid synthesis and exchange with circulating extracellular lipids. Most circulating lipids, including cholesterol and triglycerides, are packaged into lipoproteins, with the density of lipoproteins defining the circulating species. Specifically, low-density lipoprotein (LDL), deemed as “bad cholesterol,” is associated with cardiovascular disease (17). By contrast, high-density lipoprotein (HDL) is considered a “good cholesterol” due to its cardioprotective roles (18). According to the CDC, hypercholesterolemia, defined as elevated levels of LDL-cholesterol, was present in at least 86 million U.S. adults in 2020. However, the effects of circulating lipoproteins on gammaherpesvirus infection remain poorly understood.

In addition to density, lipoproteins are characterized by distinct combinations of proteins (termed apolipoproteins) that are associated with the lipids. Apolipoproteins ligate their corresponding receptors to allow cholesterol and lipid exchange in addition to activation of signaling pathways downstream of the receptor. Apolipoprotein E (ApoE) is a 34 kDa protein produced by several cell types, including hepatocytes, neurons, glia, smooth muscle cells, and macrophages (19). Though it has garnered increasing attention in recent years for its association with Alzheimer’s disease and other neurodegenerative disorders, ApoE is most known for its roles in lipid transport as a component of several lipoprotein species. ApoE is a ligand for LDL receptors and very low-density lipoprotein (VLDL) receptors to mediate the uptake of primarily cholesterol and triglycerides, respectively. ApoE also serves as a chylomicron remnant receptor ligand for dietary cholesterol and fatty acid recycling or excretion. In contrast to its role as a “bad cholesterol” receptor ligand, ApoE facilitates the process of reverse cholesterol transport from tissues to the liver through its association with HDL (20, 21). Proper function of ApoE in bidirectional lipid transport is critical for maintaining lipid homeostasis in both mice and humans. In humans, there are three isoforms of ApoE, termed E2, E3, and E4. Mice harbor a single isoform that behaves most similarly to human ApoE3, the most common and least disease-associated isoform. Importantly, genetic ApoE deficiency in both species leads to hypercholesterolemia and hypertriglyceridemia and significantly increased risk for cardiovascular disease (22–25).

Interestingly, ApoE exhibits pleiotropic roles during viral infection. ApoE interacts with envelope proteins of hepatitis C virus (HCV), hepatitis B virus (HBV), and Zika virus and incorporates into newly produced viral particles (26–30). Neutralizing ApoE or removing its cell-surface receptors attenuates entry of HCV and HBV, further implicating ApoE in supporting these viral infections. By contrast, ApoE colocalizes with the HIV-1 envelope (Env) protein and directs it to the lysosome for degradation, limiting cell-surface Env expression and attenuating HIV-1 entry (31). In terms of pathogenesis, ApoE is protective against influenza A-driven lung disease, with mice lacking one or both ApoE alleles exhibiting more severe lung pathology and greatly reduced survival (32). However, for herpes simplex virus type 1, ApoE-null mice have lower latent viral reservoirs in the central nervous system following intraperitoneal inoculation (33, 34) and lower latent reservoirs in the trigeminal ganglion following ocular infection, with a significantly improved survival compared to their wild-type counterparts (35).

Despite the existing studies of ApoE in these diverse viral infection models, the role of ApoE in gammaherpesvirus infection remains poorly understood. The only two studies that examined gammaherpesvirus-ApoE interactions demonstrated that murine gammaherpesvirus 68 (MHV68) infection accelerates the development of atherosclerosis in ApoE-deficient mice infected at weaning with a high dose of MHV68 (5 × 10^5^ PFU) (36, 37). Effects of ApoE on MHV68 replication or latency remain unknown.

This study focuses on the role of ApoE in MHV68 infection of macrophages. Macrophages are key innate immune cells that provide a first line of defense against viral infection, in addition to their role in regulating lipid homeostasis of the host. Importantly, macrophages represent a physiologically relevant cell type for gammaherpesvirus studies as they are infected by human and murine gammaherpesviruses *in vivo* during acute and chronic phases of infection, and they support lytic replication of MHV68 *in vitro* (10, 12, 38–46). We found that MHV68 infection of primary macrophages stimulated ApoE expression in a type I interferon (IFN)-dependent manner. Increased ApoE expression driven by this major antiviral host pathway was usurped by MHV68 to facilitate viral gene expression and lytic replication. By contrast, MHV68 replication was equivalent in wild-type and ApoE-deficient fibroblasts, along with significantly lower levels of ApoE expression in wild-type fibroblasts as compared to macrophages. Surprisingly, the traditional role of ApoE as a lipid regulator was not involved in the ApoE proviral effects during MHV68 lytic replication in macrophages, despite the established role of macrophages in host lipid homeostasis. Finally, ApoE was not involved in the establishment of MHV68 latent reservoir in the peritoneal cavity under conditions that enrich for latent infection of peritoneal macrophages, indicating that the proviral effects of ApoE in macrophages are likely limited to the lytic viral life cycle.

## RESULTS

### ApoE expression is increased in a type I IFN-dependent manner to support MHV68 replication in primary macrophages

**Fig 1:**
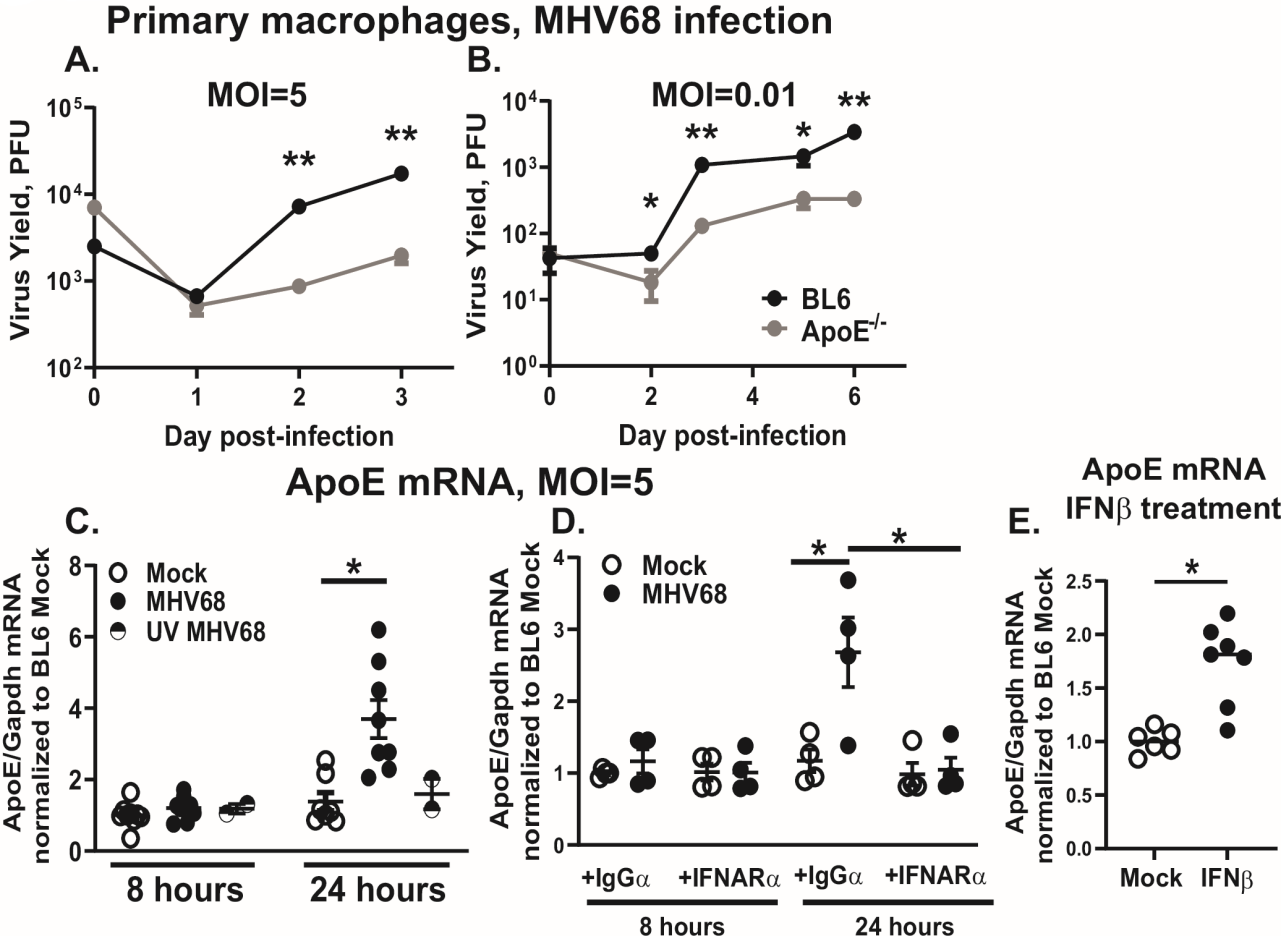
ApoE expression is increased in a type I IFN-dependent manner to support MHV68 replication in primary macrophages. (A, B) Bone marrow-derived macrophages from BL6 and ApoE^-/-^ mice were infected at the two indicated multiplicities of infection (MOI, PFU/cell). Total infectious MHV68 yield (cell-associated and supernatant) was measured at the indicated days post-infection. Data are representative of three independent experiments, and each condition was assessed in three biological replicates within each study. (**C**) BL6 macrophages were infected with live or UV-inactivated MHV68 at MOI = 5 or mock-treated. (**D**) BL6 macrophages were infected with MHV68 at MOI = 5 or mock-treated. Immediately after viral adsorption, cells were additionally treated with an antibody blocking the type I interferon receptor (IFNARα) or an isotype control (IgGα) for the duration of the experiment. (**E**) BL6 macrophages were treated with 10 U/mL of recombinant mouse IFNβ or mock-treated. (C–E) At indicated time points, total RNA was isolated and relative mRNA levels of ApoE were measured by quantitative reverse-transcription-PCR (qRT-PCR), with further normalization to the corresponding Gapdh levels. Relative levels of expression were further normalized to the average of mock-infected conditions at 8 hours post-infection (**C, D**) or mock-treated cells (**E**). Data were pooled from 2 to 4 independent experiments, with individual symbols representing biological replicates. Error bars represent the standard error of the mean (SEM). *P < 0.05, **P < 0.01 (Student’s t-test). To increase the clarity of presented data, here and in subsequent figures, only statistically significant differences are noted as such.

Given the physiological relevance of macrophages for gammaherpesvirus infection and the constitutive expression of ApoE by macrophages, MHV68 replication was assessed in BL6 and ApoE^-/-^ bone marrow-derived macrophages. Interestingly, MHV68 titers were decreased in ApoE^-/-^ macrophages at both high and low multiplicities of infection (MOI) (Fig. 1A and B), indicating that ApoE expression by macrophages supports MHV68 replication.

We previously showed that MHV68 infection of macrophages increases the expression of the low-density lipoprotein receptor (LDL-R) and liver X receptors (LXRs), important lipid metabolism regulators that attenuate MHV68 replication (10, 12). To determine the extent to which MHV68 infection affected ApoE expression, ApoE mRNA levels were assessed. ApoE mRNA levels were significantly increased in infected macrophages as compared to uninfected controls between 8 and 24 hours post-infection (Fig. 1C). The increase in ApoE mRNA was driven by active virus replication, as treatment of macrophages with UV-inactivated MHV68 did not alter baseline ApoE mRNA levels.

Interestingly, increased LDL-R and LXR expression was driven by type I IFN signaling induced in MHV68-infected macrophages (10, 12). To determine whether the increase in ApoE expression depended on type I IFN, IFN signaling was blocked by treating the macrophages (immediately after viral adsorption) with a blocking antibody against the type I IFN receptor (anti-IFNAR1) or with an isotype-matched non-specific antibody (anti-IgG) to control for the effects of Fc receptor ligation. Similar to that observed for the LDL-R and LXRs in prior studies (10, 12), blocking the type I IFN receptor returned ApoE mRNA levels in infected macrophages to baseline expression (Fig. 1D), indicating that MHV68 infection increases ApoE expression in a type I IFN-dependent manner.

To determine whether type I IFN signaling was sufficient to increase ApoE expression, ApoE mRNA levels were measured in uninfected macrophages treated with recombinant mouse IFNβ at a concentration that is observed in MHV68-infected macrophage cultures (47). ApoE mRNA levels were increased after 24 hours of IFNβ treatment (Fig. 1E), indicating that type I IFN was sufficient to stimulate ApoE expression in macrophages. In summary, ApoE expression by primary macrophages supported MHV68 replication, with ApoE expression further stimulated by active viral replication and type I IFN signaling.

### ApoE promotes MHV68 lytic gene expression in the last 24 hours of infection and viral replication downstream of viral DNA synthesis

**Fig 2:**
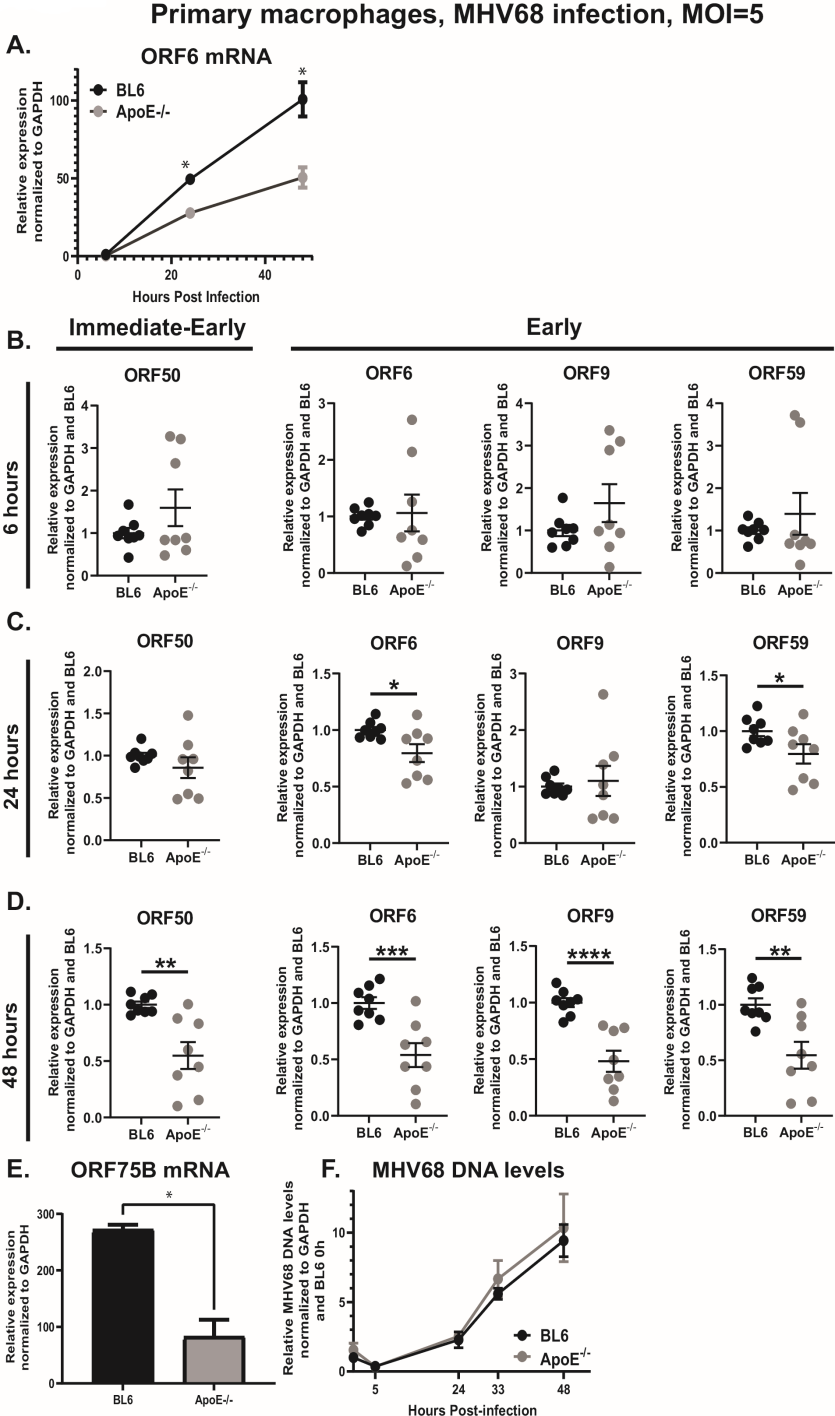
ApoE promotes MHV68 lytic gene expression in the last 24 hours of infection and viral replication downstream of viral DNA synthesis. Bone marrow-derived macrophages from BL6 and ApoE^-/-^ mice were infected at an MOI of 5 PFU/cell. (**A**). Representative experiment demonstrating relative ORF6 mRNA levels across a single replication cycle. Relative mRNA levels of ORF50, ORF6, ORF9, and ORF59 (with data pooled from three independent experiments, with individual symbols representing biological replicates) were measured by qRT-PCR at 6 hours post-infection (**B**), 24 hours post-infection (**C**), or 48 hours post-infection (**D**) with relative expression normalized to the corresponding GAPDH levels and further normalized to the average observed in BL6 MHV68-infected macrophages at each time point. (E, F). Representative data (from 2 to 3 independent experiments) demonstrating mRNA levels of ORF75B at 48 hours post-infection (**E**) or relative cell-associated MHV68 DNA levels at indicated times post-infection (**F**). Error bars represent the standard error of the mean (SEM). *P < 0.05, **P < 0.01, ***P < 0.001, ****P < 0.0001 (Student’s t-test).

Having observed that ApoE supports MHV68 lytic replication in macrophages, the effect of ApoE on viral lytic gene expression throughout the entire replication cycle was assessed next. Similar to other herpesviruses, MHV68 lytic gene expression proceeds in three phases: (i) immediate-early genes, such as the viral replication and transcription activator (RTA) encoded by ORF50; (ii) early genes, such as single-stranded DNA binding proteins (ORF6), the viral DNA polymerase (ORF9), and DNA polymerase processivity factor (ORF59); and (iii) late genes, with expression triggered by the initiation of viral DNA synthesis. A representative time course of MHV68 ORF6 expression over a single viral replication cycle is shown in Fig. 2A. Given the intrinsic intra- and inter-batch variability in bone marrow-derived primary macrophages, subsequent results (Fig. 2B through D) represent pooled data from three independent experiments, with MHV68 gene expression normalized to the average of viral gene expression observed in BL6 macrophages for each time point within each experiment.

At 6 hours post-infection, prior to the increase in ApoE expression (Fig. 1C), there was no difference in the expression of any of the assessed viral lytic genes, indicating that baseline ApoE expression by macrophages does not play a role in MHV68 entry or the initiation of viral lytic gene expression (Fig. 2B). At 24 hours post-infection, when an increase in ApoE mRNA levels was first observed, mRNA levels of ORF6 and ORF59, but not ORF50 and ORF9, were decreased in ApoE^-/-^ macrophages (Fig. 2C). By the end of the replication cycle, mRNA levels of all assessed MHV68 lytic genes were decreased in ApoE^-/-^ macrophages (Fig. 2D), including mRNA of ORF75B, a tegument protein expressed with late kinetics (Fig. 2E). Thus, the kinetics of ApoE induction in MHV68-infected macrophages temporally correlated with the proviral effects of ApoE in supporting MHV68 lytic gene expression.

MHV68 ORF50, ORF6, ORF9, and ORF59 encode proteins that are essential for MHV68 DNA synthesis, which initiates around 24 hours post-infection of primary macrophages (48). Interestingly, despite the decreased expression of MHV68 lytic genes starting at 24 hours post-infection, the accumulation of MHV68 DNA was not altered in ApoE^-/-^ macrophages (Fig. 2F), suggesting that ApoE facilitates MHV68 lytic replication downstream of viral DNA synthesis.

### ApoE deficiency does not affect type I IFN signaling in MHV68-infected macrophages

**Fig 3:**
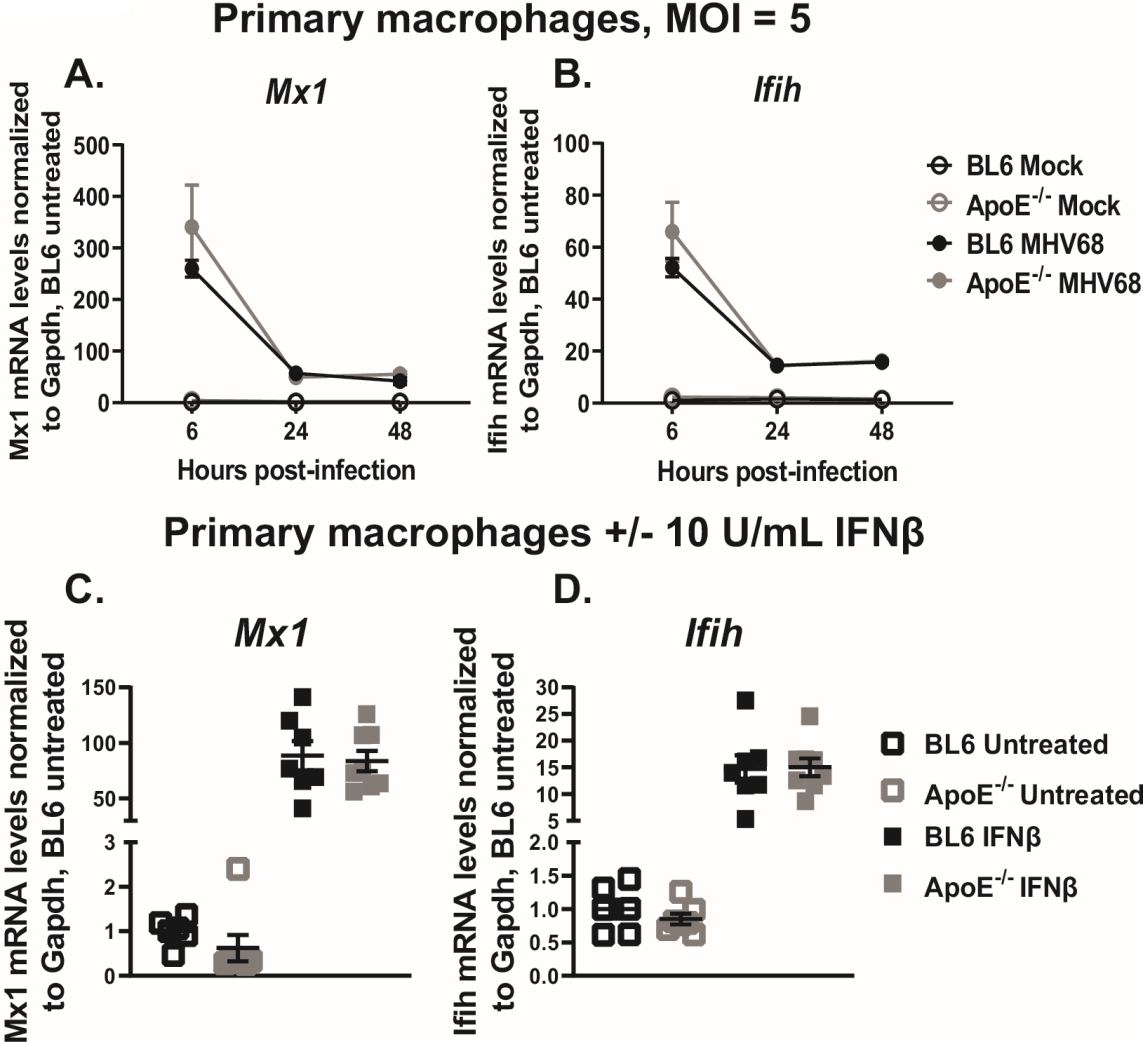
ApoE deficiency does not affect type I IFN signaling in MHV68-infected macrophages. (A, B) Bone marrow-derived macrophages from BL6 and ApoE^-/-^ mice were infected at an MOI of 5 PFU/cell or mock-treated. Relative mRNA levels of Mx1 and IFIH were measured at 6-, 24-, and 48 hours post-infection by qRT-PCR and normalized to the corresponding Gapdh mRNA levels. Data are representative of three independent experiments with three replicates/group. (C, D) Bone marrow-derived macrophages from BL6 and ApoE^-/-^ mice were treated with 10 U/mL of recombinant mouse IFNβ or mock-treated (untreated). Relative mRNA levels of Mx1 and IFIH were measured at 4 hours post-treatment by qRT-PCR, normalized to the corresponding Gapdh mRNA levels, and further normalized to the average of the BL6-untreated condition. Data were pooled from two independent experiments, with individual symbols representing biological replicates. Error bars represent the standard error of the mean (SEM).

Type I IFN signaling is critical for control of lytic MHV68 replication in macrophages (49). Given that ApoE expression is increased by type I IFN signaling and that ApoE supports MHV68 lytic replication, we next tested an intriguing hypothesis that ApoE may attenuate type I IFN signaling to exert its proviral effect. However, in contrast to our hypothesis, mRNA levels of *Mx1*, a type I IFN-dependent IFN-stimulated gene (ISG) (50), were similar in MHV68-infected BL6 and ApoE^-/-^ macrophages throughout the entire replication cycle (Fig. 3A). Similarly, the expression of *Ifih*, an ISG implicated in controlling MHV68 replication (50), was not altered by the ApoE genotype of infected macrophages (Fig. 3B). Furthermore, treatment of uninfected macrophages with recombinant IFNβ induced similar mRNA levels of *Mx1* and *Ifih* in BL6 and ApoE^-/-^ macrophages (Fig. 3C and D). In summary, ApoE expression had no effect on type I IFN signaling in primary macrophages, including during MHV68 infection.

### ApoE expression by primary macrophages does not alter the expression of lipid synthesis genes

After observing no effect of ApoE expression on type I IFN signaling in macrophages, lipid biosynthesis pathways were examined next. Endogenous synthesis of cholesterol and fatty acids supports lytic gammaherpesvirus replication, including that of MHV68 (9–14, 16). Importantly, the activity of endogenous lipid biosynthesis pathways is primarily regulated by the transcription of the involved enzymes and is counterbalanced by the uptake of extracellular lipids and efflux of intracellular lipids to achieve lipid homeostasis. At the cellular level, particularly for macrophages, ApoE mediates both lipid uptake through its activity as an LDL-R ligand and cholesterol efflux from the cell to HDL via its associations with scavenger receptor class B type I (SR-BI) and ATP binding cassette, subfamily A member 1 (ABCA1) and subfamily G member 1 (ABCG1) (51–55). However, the role of ApoE in stimulating or attenuating endogenous lipid synthesis in macrophages is poorly understood.

**Fig 4:**
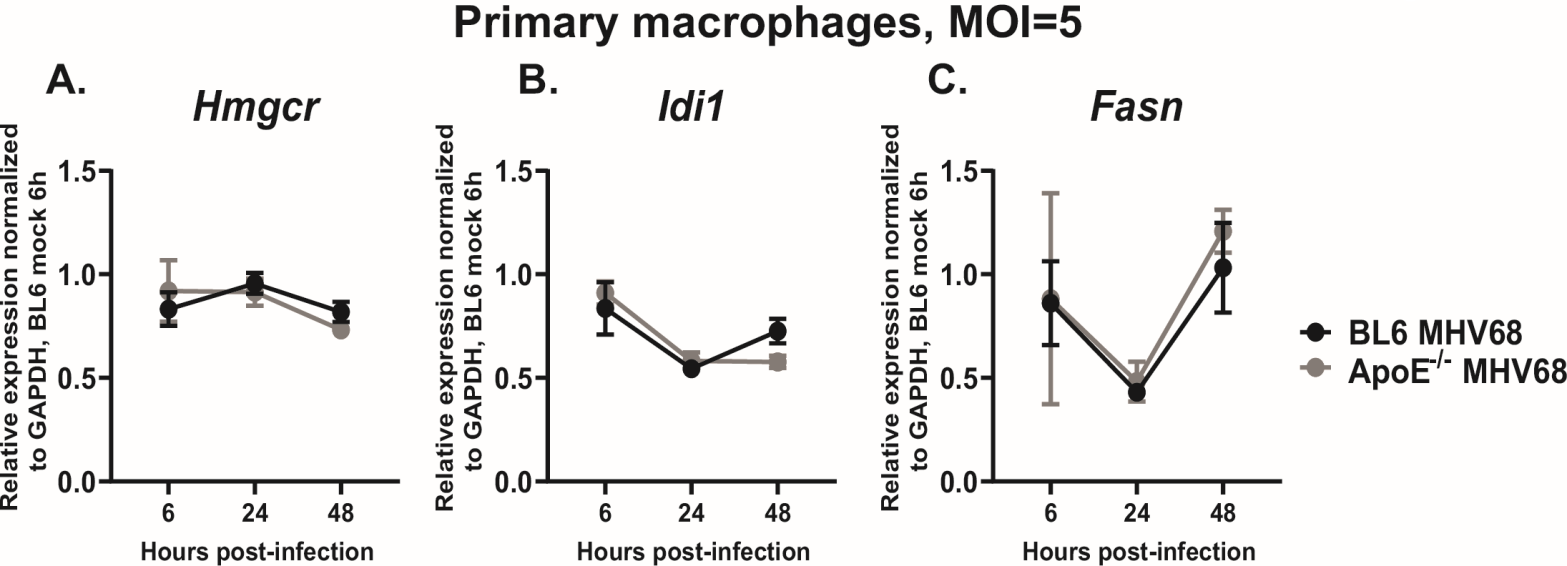
ApoE expression by primary macrophages does not alter expression of lipid synthesis genes. Bone marrow-derived macrophages from BL6 and ApoE^-/-^ mice were infected at an MOI of 5 PFU/cell or mock-treated. Relative mRNA levels of (**A**) Hmgcr, (**B**) Idi1, and (**C**) Fasn were measured at 6-, 24-, and 48 hours post-infection by qRT-PCR, normalized to the corresponding Gapdh mRNA levels and further normalized to relative levels of expression in the mock-treated condition (set to 1). Data were pooled from two independent experiments with three replicates/group, and values from infected groups only are shown for figure clarity. Error bars represent the standard error of the mean (SEM).

Thus, to determine the extent to which ApoE expression by MHV68-infected primary macrophages altered expression of fatty acid and cholesterol synthesis enzymes, mRNA levels of relevant enzymes were assessed throughout the MHV68 replication cycle in BL6 or ApoE^-/-^ macrophages. The mRNA levels of 3-hydroxy-3-methylglutaryl-coenzyme A reductase (*Hmgcr*) and isopentenyl-diphosphate delta isomerase (*Idi1*), two key enzymes involved in cholesterol synthesis, were similar in BL6 or ApoE^-/-^ macrophages at all examined time points throughout the MHV68 lytic cycle (Fig. 4A and B). Similarly, mRNA levels of fatty acid synthase (*Fasn*) were not affected by the ApoE genotype of infected macrophages (Fig. 4C). In summary, expression of ApoE by MHV68-infected macrophages did not affect expression of fatty acid or cholesterol synthesis genes.

### ApoE expression by fibroblasts does not promote MHV68 replication

**Fig 5:**
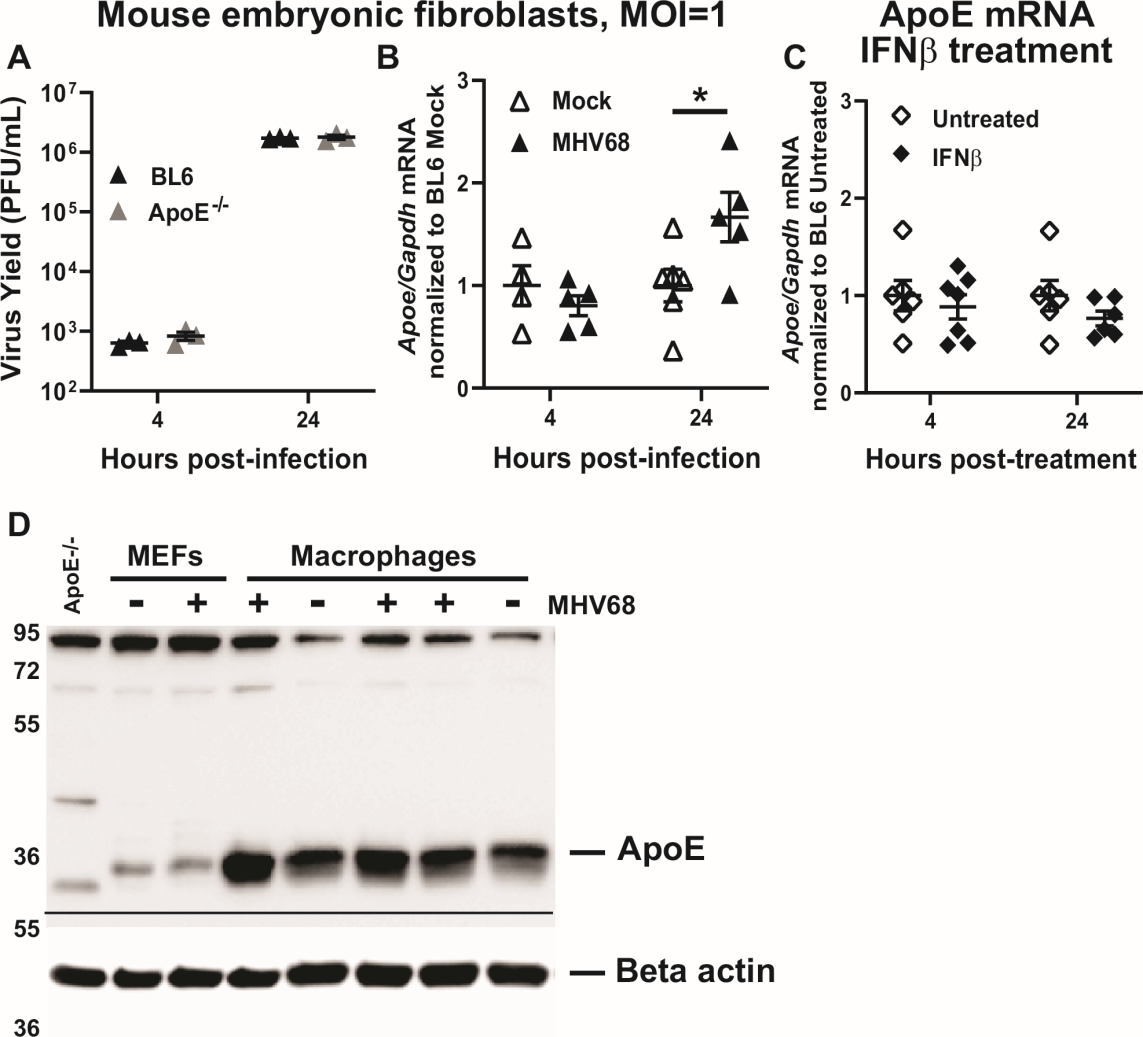
ApoE expression by fibroblasts does not promote MHV68 replication. (**A**) Mouse embryonic fibroblasts (MEFs) derived from BL6 and ApoE^-/-^ mice were infected at an MOI of 1 PFU/cell, and total infectious MHV68 yield was measured as in Fig. 1 at the indicated hours post-infection. Data represent two independent experiments, with individual symbols representing biological replicates. (**B**) BL6 MEFs were infected at MOI = 1 or mock-treated. Relative mRNA levels of ApoE were measured by qRT-PCR with normalization to Gapdh and further normalized to relative levels of expression in the mock-treated condition (set to 1). (**C**) BL6 MEFs were treated with 10 U/mL of recombinant mouse IFNβ or mock-treated (untreated). Relative mRNA levels of ApoE were measured by qRT-PCR with normalization to Gapdh and further normalized to relative levels of expression in the untreated condition. Data were pooled from two independent experiments, with individual symbols representing biological replicates. Error bars represent the standard error of the mean (SEM). *P < 0.05 (Student’s t-test). (**D**). Macrophages or MEFs were mock-treated or infected at an MOI of 5 or 1, respectively. Brefeldin A was added at 10 µg/mL to the existing culture medium at 18 hours post-infection. Cell lysates collected at 24 hours post-infection were subjected to western analysis using indicated antibodies. Each macrophage sample represents an independent biological replicate. Cell lysate from uninfected ApoE^-/-^ macrophages was used as an antibody specificity control. Numbers represent molecular weight in kDa. The line indicates that independent gels were used to measure ApoE and beta actin levels.

Macrophages have a well-established role in the regulation of lipid homeostasis of the host in contrast to most other immune and somatic cell types, including fibroblasts. Furthermore, we previously showed that the antiviral roles of LXRs and LDL-R were selective for MHV68-infected primary macrophages, but not mouse embryonic fibroblasts (MEFs) (10, 12). Therefore, the cell type-specific role of ApoE in MHV68 replication was tested in BL6 and ApoE^-/-^ MEFs. In contrast to that observed for macrophages, MHV68 replication was similar in BL6 and ApoE^-/-^ MEFs (Fig. 5A). Interestingly, ApoE mRNA levels were modestly increased in MHV68-infected MEFs by 24 hours post-infection (Fig. 5B), a time point that corresponds to the completion of a single MHV68 replication cycle in MEFs (16–24 hours at MOI = 1). Furthermore, unlike that seen in primary macrophages, treatment with recombinant type I IFN was not sufficient to induce ApoE expression in MEFs (Fig. 5C). Therefore, despite observing an increase in ApoE expression late in the MHV68 replication cycle, MHV68 lytic replication was not affected by the ApoE genotype of MEFs.

Macrophages express robust levels of ApoE, consistent with their role in regulation of host lipid homeostasis. While the expression of ApoE by fibroblasts remains less defined, human foreskin fibroblasts were reported to express ApoE at baseline (56). Given the presence of bovine ApoE in the tissue culture medium that precluded accurate measurement of secreted mouse ApoE, expression of endogenous mouse ApoE was compared in mock- or MHV68-infected primary macrophages and MEFs that were treated with brefeldin A 18–24 hours post-infection to prevent protein secretion, followed by western analyses of cell lysates. As expected, MHV68-infected macrophages demonstrated an increase in ApoE protein levels following MHV68 infection (Fig. 5D). By contrast, ApoE expression was significantly lower in fibroblasts, as compared to macrophages, with no appreciable increase seen in infected cells (Fig. 5D). Thus, very low expression of ApoE observed in fibroblasts, as compared to macrophages, was likely responsible for the similar MHV68 replication observed in BL6 and ApoE^-/-^ MEFs.

### ApoE deficiency does not affect the establishment of MHV68 latency under conditions that enrich for macrophages as a latent reservoir *in vivo*

**Fig 6:**
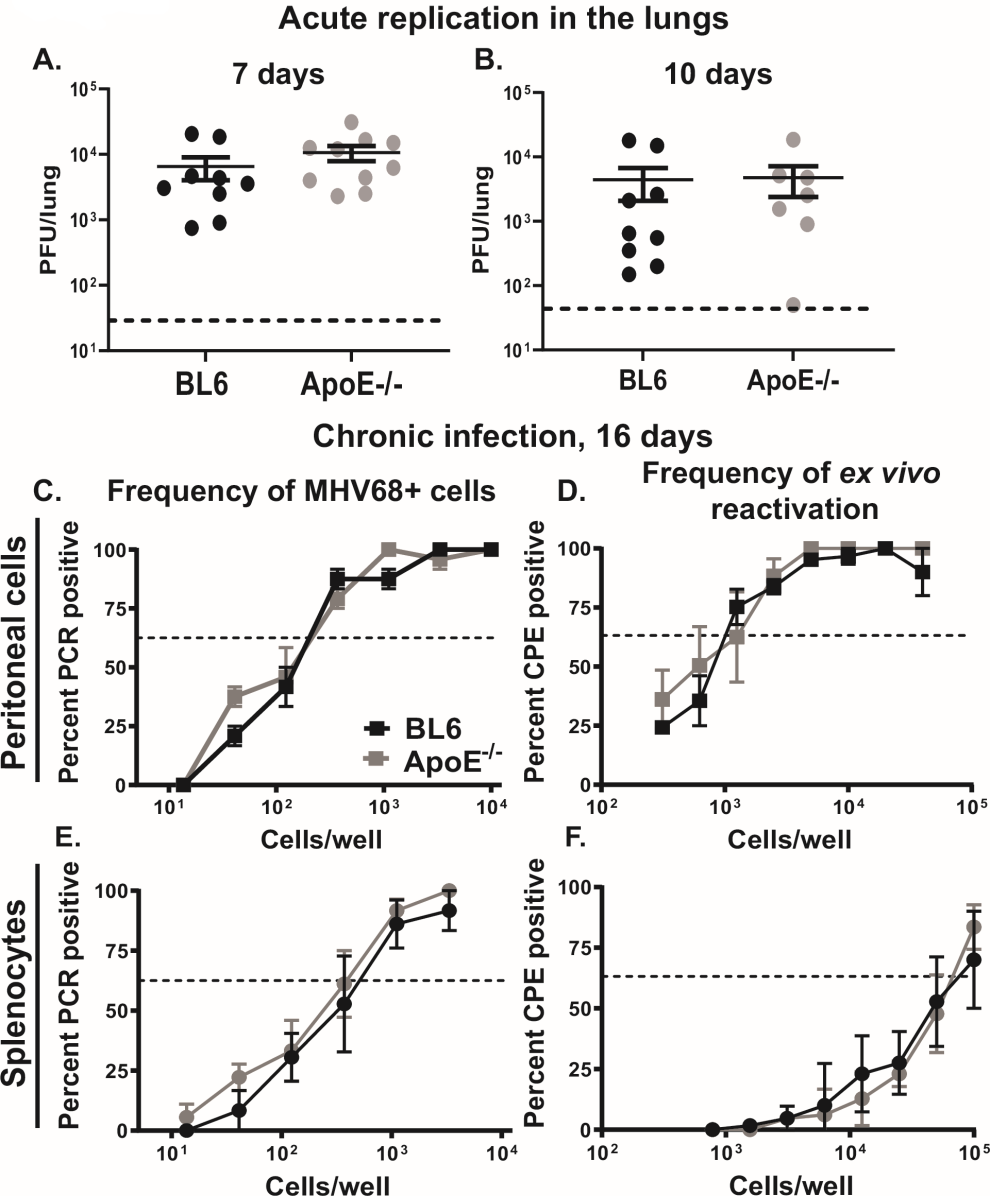
ApoE deficiency does not affect the establishment of MHV68 latency under conditions that enrich for macrophages as a latent reservoir *in vivo*. (A, B) BL6 and ApoE^-/-^ mice were inoculated with 1,000 PFU of MHV68 intranasally. Lung MHV68 titers were measured at 7 (**A**) or 10 (**B**) days post-infection. Each symbol represents an individual mouse. (C–F) BL6 and ApoE^-/-^ mice were inoculated with 1,000 PFU of MHV68 by intraperitoneal injection. At 16 days post-infection, peritoneal cells (C, D) and total splenocytes (E, F) were harvested and subjected to limiting dilution analyses to determine the frequency of latently infected cells (C, E) or the frequency of *ex vivo* reactivation (D, F). Prior to limiting dilution analyses, peritoneal cells or splenocytes from 3 to 5 mice/group were pooled within each study; pooled data from three independent experiments are shown. In the limiting dilution assays, the dotted line is drawn at 63.2% and the x-coordinate of the intersection of this line with the sigmoid graph represents an inverse of the frequency of positive events. Error bars represent the standard error of the mean (SEM).

The proviral role of ApoE during lytic MHV68 replication identified in primary macrophages prompted the investigation of the role of ApoE during acute MHV68 replication *in vivo*. Macrophages and epithelial cells support acute MHV68 replication in the lungs following intranasal inoculation. Importantly, epithelial cells represent the majority of infected cells in the lung of an immunocompetent animal during acute infection (57). Consistent with macrophages representing a minority of acutely infected lung cells and the cell type-specific proviral role of ApoE we observed *in vitro* (Fig. 1A, B, and 5), there was no difference in MHV68 lung titers observed in BL6 and ApoE^-/-^ mice at the peak (7 days, Fig. 6A) or clearance (10 days, Fig. 6B) phases of acute MHV68 infection *in vivo*.

Following intranasal inoculation, the latent MHV68 reservoir is hosted primarily in B cells in both the spleen and peritoneal cavity (40, 58, 59). However, the intraperitoneal inoculation route, while supporting latent infection of splenic B cells, allows MHV68 to preferentially establish latency in peritoneal macrophages, at least at 16 days post-infection (40). To determine whether ApoE supports latent MHV68 infection of macrophages during chronic infection, BL6 and ApoE^-/-^ mice were infected intraperitoneally, and frequencies of latently infected cells and *ex vivo* reactivation were assessed using limiting dilution assays. Interestingly, the frequencies of latently infected peritoneal cells and MHV68 *ex vivo* reactivation were similar in BL6 and ApoE^-/-^ mice at 16 days post-intraperitoneal MHV68 inoculation (Fig. 6C and D). Furthermore, the frequencies of latently infected splenocytes and *ex vivo* MHV68 reactivation in the spleens were also similar in BL6 and ApoE^-/-^ mice, indicating that ApoE expression does not regulate the establishment of chronic MHV68 infection following the route of inoculation that enriches for latent reservoir in the peritoneal macrophages.

## DISCUSSION

Our study defines a novel cell type- and viral life cycle-specific proviral role of ApoE during gammaherpesvirus infection. We demonstrate that ApoE expression by primary macrophages does not affect early stages of MHV68 lytic replication, unlike that previously shown for HBV, HCV, Zika, and HIV (26, 27, 31). By contrast, ApoE expression, which is further enhanced by type I IFN signaling in infected primary macrophages, is usurped by MHV68 to facilitate viral lytic gene expression during the second half of the replication cycle. Our studies rule out the involvement of type I IFN signaling or endogenous lipid synthesis pathways in the observed proviral ApoE effects, suggesting that novel roles of ApoE are responsible for the observed proviral effects. Finally, ApoE deficiency did not affect the establishment of chronic MHV68 infection under conditions that enrich for latent infection of peritoneal macrophages, suggesting that the proviral effects of ApoE are limited to the lytic viral cycle.

We found that endogenous ApoE expression by macrophages supported expression of MHV68 lytic genes of all three kinetic classes (immediate early, early, and late). Importantly, the proviral effects of ApoE on lytic gene expression were limited to the second half of the replication cycle, without affecting the initiation of lytic gene expression or viral DNA synthesis. Given these findings, ApoE genetic status of primary macrophages did not affect MHV68 entry. While it is possible that ApoE could be incorporated into the MHV68 virion, similar to that observed for select RNA viruses (26–30), we showed that genetic deficiency of LDL-R, a high-affinity ApoE receptor, had no effect on MHV68 entry into primary macrophages. Instead, LDL-R functioned to attenuate MHV68 lytic gene expression (12). Given that ApoE is a secreted protein, its proviral effects on MHV68 gene transcription must be a result of either metabolic changes or signaling pathways engaged downstream of ApoE receptors other than LDL-R.

Type I IFN signaling and endogenous lipid synthesis were pursued first as potential mechanistic explanations for ApoE’s proviral role, given the critical importance of type I IFN in attenuating MHV68 replication and the well-established roles of ApoE in lipid metabolism (10, 11, 60). Having ruled out the involvement of these host processes in the proviral role of ApoE, future studies must consider additional ApoE-dependent mechanisms. Intriguingly, ApoE attenuates acute inflammatory signaling pathways that lead to activation of nuclear factor-kappa B (NF-kB) in macrophages and *in vivo* (61–64). However, the role of NF-kB in MHV68 lytic replication remains controversial. One study demonstrated that active p65 attenuates MHV68 lytic replication in HEK 293T cells (65). By contrast, deficiency of mitochondrial antiviral signaling (MAVS) upstream of NF-kB activation results in decreased MHV68 replication in MEFs (66). Finally, the expression of constitutively active NF-kB inhibitor by MHV68 did not affect viral replication in MEFs or during acute infection *in vivo* (67). Thus, it is unclear whether ApoE-dependent attenuation of NF-kB activity in MHV68-infected macrophages (if it indeed occurs) plays into the observed viral phenotypes. Similarly, human ApoE2, 3, and 4 all activate ERK in primary human neurons (68). While ERK expression and activity support MHV68 replication in fibroblast cell line (69), the proviral role of ERK in MHV68 replication remains to be confirmed in macrophages, including in the context of ApoE deficiency.

Interestingly, despite decreased MHV68 replication in ApoE^-/-^ primary macrophages, the acute MHV68 lung titers were similar in BL6 and ApoE^-/-^ mice. MHV68 replication in the lung is primarily supported by lung epithelial cells, with a smaller proportion of infected cells represented by alveolar macrophages (57). Lung epithelial cells have not been described to express ApoE, and it is possible that if a decrease in lytic MHV68 titers does selectively occur in macrophages of ApoE^-/-^ lungs, this would lead to a minimal, if any, effect on the overall lytic titers that are largely supported by infected epithelial cells. Importantly, using a route of inoculation that enriches for latent infection of peritoneal macrophages (40), our study indicates that the proviral role of ApoE is selective for the lytic and not latent MHV68 life cycle. This is perhaps not that surprising, as the host mechanisms that regulate lytic vs. latent infection only partially overlap, as shown by our and other groups (11, 46, 67, 70). It is also important to note that young mice (6–7 weeks of age) were exclusively used for *in vivo* infections in this study to exclude the confounding effects of atherosclerosis that spontaneously develops in ApoE^-/-^ mice by 3 months of age, even when maintained on a standard chow diet (23). The results of the current study provide a foundation for future studies that will define the potential effects of progressive atherosclerosis in aged ApoE^-/-^ mice on the control of long-term MHV68 infection, as well as the selective effect of MHV68 infection in accelerating atherosclerosis in this mouse strain. Interestingly, despite a lack of overt atherosclerosis, young ApoE^-/-^ mice demonstrate increased levels of LDL-cholesterol and serum triglycerides (71) that do not change following MHV68 infection (data not shown). However, the establishment of latent MHV68 reservoir at 16 days post-infection was not affected by the ApoE genotype of the mice in this study (Fig. 6). This lack of ApoE-dependent phenotype suggests that either elevated plasma cholesterol and triglycerides have no effect on MHV68 infection of an intact host (in contrast to that observed for acute influenza and SARS-CoV-2 infections in high-fat diet-fed mice [72, 73]) or that ApoE engagement of corresponding receptors in the presence of increased circulating lipids may be required to alter parameters of chronic MHV68 infection. These hypotheses will be tested in future studies of diet-based manipulation of circulating cholesterol and lipid levels.

## MATERIALS AND METHODS

### Animal studies and primary cell cultures

C57BL/6J (BL6) and ApoE^-/-^ mice (23) were obtained from The Jackson Laboratory (Bar Harbor, ME) and bred and housed in a specific-pathogen-free barrier facility at the Medical College of Wisconsin (MCW). For acute infection studies, male and female 6- to 7-week-old mice were inoculated intranasally with 1,000 PFU of MHV68 (WUMS strain) under light anesthesia. Viral titers were determined by plaque assay on NIH 3T12 fibroblasts from lung homogenates as previously described (59). For chronic infection studies, 6- to 7-week-old mice were inoculated intraperitoneally with 1,000 PFU of MHV68 in a volume of 300 µL of Dulbecco’s modification of Eagle’s medium (Corning). Virus stocks were prepared and titered on NIH 3T12 cells (74).

Bone marrow macrophages were derived from male and female 3- to 4-week-old mice as described previously (74), with each experiment using sex- and age-matched mice. Mouse embryonic fibroblasts (MEFs) were isolated from embryonic days 12–14, and early passage (p1-4) MEFs were used for all experiments. Passage-matched MEFs were used for all experiments comparing BL6 and ApoE^-/-^ genotypes.

### Viral infections and cell treatments *in vitro*

Bone marrow-derived macrophages and MEFs were exposed to live virus in a small volume of media for 1 h at 37°C, followed by three washes with PBS and replacement of the full volume of primary cell media. Day 0 titers for viral growth curves are generated from samples where macrophages were scraped into the media immediately following media replacement and represent a combination of extracellular virus not removed by washes, infectious virions associated with macrophage cell surface, and intracellular infectious virions prior to lysosomal digestion or productive infection. For the IFNAR-blocking experiments in Fig. 1, anti-IFNAR1 antibody or anti-IgG isotype control was added with media replacement after viral adsorption. Both antibodies were used at 500 ng/mL and purchased from Bio X Cell (Lebanon, NH). Recombinant mouse interferon beta (IFNβ) was purchased from BioLegend (San Diego, CA) and used at a concentration of 10 units/mL (U/mL). For brefeldin A treatment, macrophages or MEFs were infected as above, and brefeldin A (Cayman Chemical, Ann Arbor, MI) was added to the media (10 µg/mL) at 18 hours post-infection, with cell lysates prepared at 24 hours post-infection, as described below.

### qRT-PCR

Total RNA from bone marrow-derived macrophages and MEFs was harvested, subjected to DNAse treatment and reverse transcription, and analyzed by quantitative reverse transcription-PCR (qRT-PCR) (47). Relative expression was quantified by the delta-delta threshold cycle with *Gapdh* used as a housekeeping gene. The following primers were used in qRT-PCR analysis:

*Gapdh* Forward (5′-TGTGATGGGTGTGAACCACGAGAA-3′); *Gapdh* Reverse (5′-GAGCCCTTCCACAATGCCAAAGTT-3′); *Apoe* Forward (5′-GGCAAACCTGATGGAGAAGATA-3′); *Apoe* Reverse (5′-TTGTTGCAGGACAGGAGAAG-3′); ORF50 Forward (5′-AGAAACCCACAGCTCGCACTT-3′); ORF50 Reverse (5′-CAATATGCTGGACAGGCGTATC-3′); ORF6 Forward (5′-GTTGCCAGATATCCCTAGGATGA-3′); ORF6 Reverse (5′-ACCTGGCTGGGTCAAGAGACT-3′); ORF9 Forward (5′-TGCATGCAAGTTTGTCCAGTCT-3′); ORF9 Reverse (5′-CTTCCCCCAGTTACTCATTGTTTG-3′); ORF59 Forward (5′-TGACTGGCAGGTTTTTGTATGC-3′); ORF59 Reverse (5′-GATGATCGTGAGGCCAATGG-3′); ORF75B Forward (5′-CAGGGCAGGTGTTCAGATTT-3′); ORF75B Reverse (5′-CTTCGGAGATGGTCAACAGG-3′); *Mx1* Forward (5′-AGCTAGACAGAGCAAACCAAGCCA-3′); *Mx1* Reverse (5′-TCCCTGAAGCAGACACAGCTGAAA-3′); *Ifih* Forward (5′-GGAGTGGACAATGGCACAACATCA-3′); *Ifih* Reverse (5′-TGACGAAACTGTGGTCTCCACACA-3′); *Hmgcr* Forward (5′-GTGTCCCGTATAGCACAGCA-3′); *Hmgcr* Reverse (5′-AGGCATATCGCCCAAACCAT-3′); *Idi1* Forward (5′-GCCAGCAACAACCAGAATTT-3′); *Idi1* Reverse (5′-GTATGTTTCCTCAGCCCTACTC-3′); *Fasn* Forward: (5′-CCCCTCTGTTAATTGGCTCC-3′); *Fasn* Reverse: (5′-TTGTGGAAGTGCAGGTTAGG-3′).

### Limiting dilution assays

The frequency of MHV68-DNA-positive cells and frequency of *ex vivo* reactivation of MHV68 were determined as previously described (75). Briefly, to determine the frequency of cells harboring viral DNA, splenocytes and peritoneal exudate cells were isolated and pooled from all mice within each experimental group (3–5 mice/group), and 6 serial threefold dilutions were subjected to a nested PCR reaction (12 replicates/dilution) using primers against the viral genome. To determine the frequency of cells reactivating virus *ex vivo*, splenocytes or peritoneal exudate cells were pooled from all mice within each experimental group (3–5 mice/group), and 8–12 serial twofold dilutions of cell suspensions from each group were plated onto monolayers of mouse embryonic fibroblasts (MEFs) at 24 replicates per dilution. Cytopathic clearing of MEFs was scored at 21 days post-plating. The use of primary MEFs to amplify virus lowers the sensitivity of lytic MHV68 detection below a single plaque-forming unit.

### Viral DNA accumulation

Total DNA was isolated from infected macrophages at indicated time points, and MHV68 DNA was measured by real-time PCR, as described in (10). ORF59 primers were used to measure MHV68 DNA, with results normalized to the corresponding GAPDH levels.

### Western blot analyses

Following brefeldin A treatment, mock- and MHV68-infected macrophages or MEFs were washed on ice at least three times with cold PBS to eliminate contamination from bovine ApoE. After PBS was removed, cells were lysed in radioimmunoprecipitation assay (RIPA) buffer containing protease inhibitors (1 µg/mL pepstatin/leupeptin/aprotinin and 20 µg/mL phenylmethylsulfonyl fluoride), and total protein concentrations were determined by the Lowry method. Cellular lysates (30 µg protein) were separated by 10% SDS-PAGE and wet-transferred to nitrocellulose membranes. The membranes were blocked with 5% nonfat milk (wt/vol) in 1× Tris-buffered saline with 0.05% Tween-20 (TBST) at room temperature for 1 hour and then probed with anti-ApoE (1:1,000, Abcam, Cambridge, UK, ab83115) and anti-beta actin (1:2,500, Thermo Fisher Scientific, Waltham, MA, AM4302) antibodies in 1% milk in TBST overnight at 4°C. Membranes were washed and incubated with horseradish peroxidase (HRP)-conjugated anti-rabbit-IgG or HRP-conjugated anti-mouse-IgG secondary antibody (1:10,000) in 1% milk in TBST for 1 hour at room temperature. The membranes were washed, and SuperSignal West Pico PLUS (Thermo Fisher Scientific, Waltham, MA) chemiluminescent substrate was allowed to react with the membranes for 5 minutes before blots were imaged using a ChemiDoc MP.

## Data Availability

No large data sets were generated in the presented study. All data associated with this study are presented in the figures.
